# Prevalence and Predictors of Hepatitis B Virus Infection Among Sellers and Workers at West Africa′s Largest Market, Kejetia, Ghana: A Cross‐Sectional Study

**DOI:** 10.1155/bmri/1412674

**Published:** 2026-09-23

**Authors:** De-Graft Kwaku Ofosu Boateng, Michael Agyemang Obeng, Joshua Kofi Attah, Abdul Latif Koney Shardow, Clinton Owusu Boateng, Daniel Kobina Okwan, Senam Yawa Nunamey Ahadzie, Pius Takyi, Godfred Yawson Scott, Akwasi Amponsah Abrampah, Felix Kusi Boakye, Patrick Oduro, Daniel Dzemedo Nouwati, Kofi Bonsu Amankwah, Reem Mahmoud Hassanin, Mohammed Mahi Adams, Abu Abudu Rahamani

**Affiliations:** ^1^ Department of Pathology, Elbe Kliniken Stade-Buxtehude, Stade, Germany; ^2^ Kumasi Centre for Collaborative Research in Tropical Medicine, Kwame Nkrumah University of Science and Technology, Kumasi, Ghana, knust.edu.gh; ^3^ Pathology Department, Komfo Anokye Teaching Hospital, Kumasi, Ghana, kathhsp.org; ^4^ Department of Anatomy, Kwame Nkrumah University of Science and Technology, Kumasi, Ghana, knust.edu.gh; ^5^ Department of Medical Diagnostics, Kwame Nkrumah University of Science and Technology, Kumasi, Ghana, knust.edu.gh; ^6^ Dawurampong Polyclinic, Ghana Health Service, Dawurampong, Ghana, ghanahealthservice.org; ^7^ Maternal and Child Health Hospital, Ghana Health Service, Kumasi, Ghana, ghanahealthservice.org; ^8^ Public Health, Hamburg University of Applied Sciences, Hamburg, Germany, haw-hamburg.de; ^9^ National Cardiothoracic Center, Korle Bu Teaching Hospital, Accra, Ghana, kbth.gov.gh; ^10^ Department of Medical Laboratory Sciences, University for Development Studies, Tamale, Ghana, uds.edu.gh

**Keywords:** cross-sectional study, Ghana, HBsAg prevalence, Hepatitis B virus, informal sector workers, Kejetia Market, predictors, public health, vaccination

## Abstract

**Background:**

Hepatitis B virus (HBV) infection remains a major public health challenge in sub‐Saharan Africa, including Ghana, despite the infant vaccination programs. Limited data exist on HBV prevalence and predictors in informal sector populations, who may face unique occupational and behavioral exposures. This study assessed the prevalence and predictors of HBV infection among sellers and workers at Kejetia Market, Ghana′s largest commercial hub.

**Methods:**

A cross‐sectional study was conducted among 489 adult market workers from December 4, 2024, to January 25, 2025. Participants were selected through stratified random sampling across occupational groups. Data on sociodemographic, occupational, and behavioral factors were collected using structured questionnaires. On‐site testing for Hepatitis B surface antigen (HBsAg) was performed using the Hightop One Step Rapid Test kit. Bivariate and multivariate logistic regression analyses were used to identify independent predictors of HBV infection. *p* < 0.05 was considered statistically significant.

**Results:**

The overall prevalence of HBV infection was 7.36% (36/489), consistent with intermediate‐to‐high endemicity. Multivariate analysis identified three independent predictors of HBV infection. Female gender (aOR = 0.455, 95% CI: 0.221–0.937; *p* = 0.033) and absence of tattoos (aOR = 0.283, 95% CI: 0.110–0.730; *p* = 0.009) were associated with lower risk of HBV infection, whereas unvaccinated individuals had 3.37‐fold increased odds of getting the infection (95% CI: 1.395–8.142; *p* = 0.007). HBV prevalence declined progressively with increasing vaccine doses, from 9.2% in unvaccinated individuals to 2.3% among those who had completed three or more doses.

**Conclusion:**

HBV infection is common among Kejetia Market workers, with prevalence exceeding both continental and global estimates. Gender, tattooing, and vaccination status were significant predictors of infection. These findings highlight the urgent need for targeted adult Hepatitis B vaccination programs and strengthened occupational health interventions. They also emphasize the importance of regulating high‐risk practices such as informal tattooing within Ghana′s informal sector to reduce HBV burden and achieve Ghana′s contribution to the WHO goal of eliminating HBV by 2030.

## 1. Introduction

Hepatitis B virus (HBV) infection affects 296 million people worldwide, accounting for approximately 1.1 million deaths annually, mainly due to cirrhosis and hepatocellular carcinoma [1, 2]. This burden is disproportionately concentrated in the Western Pacific and sub‐Saharan Africa (SSA) [3]. Ghana illustrates this with an overall HBV prevalence of 8.36% among adults, underscoring the country′s high endemic status by World Health Organization (WHO) categorization [4].

Preventive efforts in Ghana have mostly targeted infants, with the introduction of a pentavalent vaccine in 2002 that combines HBV with other essential infectious disease vaccines into the national immunization program [5]. This drive can be attributed to the significant reduction to 2% overall HBV prevalence among children in Ghana and Senegal [6]. Despite this effort, HBV remains a significant public health issue in Ghana among the adult populace [4, 7–9].

Several factors influence HBV infection in Ghana. Body modifications (scarification, tattooing, piercing, and circumcision) are critical infection pathways, as well as exposure to infected blood and saliva, reuse and sharing of sharp objects and personal items, sexual activities, and lack of vaccination [6, 10, 11].

Occupational factors play an essential role in HBV infection and transmission risk among specific population groups. Previous studies have shown that specific occupational groups such as barbers and long‐distance drivers exhibit higher HBV prevalence (14.40%), suggesting the inherent risk of infection associated with certain professions and their occupational environment [4]. These findings suggest that certain workplace exposures, safety practices, and access to preventive measures can influence the infection risk [12]. It is thus relevant to understand the role occupational and behavioral factors play in HBV prevalence.

Previous studies on HBV in Ghana have focused on pregnant women, blood donors, and the clinical settings [4, 13]. However, there is a scarcity of data focusing on prevalence and predictors of HBV infection within the nonclinical setting especially among informal sector workers in high‐traffic commercial hubs like the Kejetia Market.

Kejetia Market is one of the largest and busiest commercial hubs in West Africa with over 20,000 vendors. These workers may be at increased risk of HBV infection because of their work environment, including factors such as close interpersonal interactions and frequent exchange of goods, frequent exposure to sharp objects among specific occupations, and potential close contact with infected individuals [14, 15]. To our knowledge, this is the first large‐scale study assessing HBV prevalence and predictors among informal market workers in Ghana, a population often excluded from routine surveillance. The market′s pivotal role in the regional economy suggests that health interventions in this target population could possess sweeping benefits.

This study is aimed at assessing the prevalence and predictors of HBV infection among sellers and workers at the Kejetia Market. Specifically, we sought to identify the sociodemographic and behavioral factors that may predispose this population to HBV infection.

This research addresses critical gaps in understanding HBV dynamics in the informal work settings. Findings will inform context‐specific prevention strategies, resource allocation, and policy guiding decisions on the National Viral Hepatitis Control Programme (NVHCP), contributing to achieving the Sustainable Development Goal (SDG) of reducing Hepatitis B mortality by 65% by 2030 [16]. Given the prevalence of similar commercial environments throughout SSA, insights from this study may have broad regional relevance for HBV prevention efforts across the continent.

## 2. Materials and Methods

### 2.1. Study Design

A cross‐sectional study design was employed to assess the prevalence and determinants of HBV infection among sellers and workers at Kejetia Market, Kumasi, Ghana, from December 4, 2024, to January 25, 2025. This was done as it allowed for the simultaneous assessment of HBV status and its associated risk factors at a specific point in time within the target population. The preprint version of the manuscript can be found online [17].

### 2.2. Study Setting and Population

The study was conducted at the Kejetia Market, Kumasi, one of the largest commercial hubs in West Africa. With over 8000 stores and stalls and about 20,000 vendors, the market hosts diverse range of occupational groups engaged in daily activities [14, 15]. The target population comprised adult sellers and workers (≥ 18 years) across various occupational categories, including butchers, fishmongers, tailors, head porters, drivers, food vendors, and general goods storekeepers. These occupational groups were selected because of their potential differential exposure to HBV risk factors, including occupational injuries, close interpersonal interactions, personal hygiene practices, and varying levels of healthcare access.

### 2.3. Sampling Technique

A total of 489 participants were recruited using a stratified random sampling technique to ensure adequate representation of different occupational groups within Kejetia Market. The market population was first categorized into predefined strata based on work type. Within each occupational stratum, participants were randomly selected using a systematic random sampling approach where every nth individual was selected after identifying a random starting point.

The number of workers in each occupational category was obtained from the available registers and subsequently verified through discussions with section leaders and market executives. Based on the size of each occupational group in relation to the total eligible population, proportional allocation was used to determine the number of participants to be recruited from each stratum. Participants within the respective strata were then selected through simple random sampling. This sampling strategy enhanced the representativeness of the study population, reduced the likelihood of selection bias, and ensured that the distribution of participants broadly reflected the occupational structure of informal workers at Kejetia Market.

The minimum sample size was calculated to be 377 participants using the Raosoft sample size calculator at 95% confidence level, a 5% margin of error, an estimated population size of 20,000, and a response distribution of 50% (to maximize sample size) [18]. The total sample size was increased to 489 to improve statistical power.

### 2.4. Inclusion and Exclusion Criteria

The study participants were adult sellers and workers (≥ 18 years) who gave written informed consent and had worked at the site for a minimum of 6 months. Persons who were incapable of providing informed consent because of a mental health disorder were excluded.

### 2.5. Data Collection

A well‐structured questionnaire was pretested, validated, and administered through face‐to‐face interviews with the participants to collect data on their sociodemographic, occupational, HBV vaccination status, and behavioral factors.

Data collection was conducted from December 4, 2024, to January 25, 2025, by trained research assistants proficient in local languages. Before commencement of the study, all researchers underwent training and retraining prior to the pretesting of the questionnaire. After each day of data collection, a cadre of investigators meticulously reviewed the data obtained, checking for inconsistencies and omissions. The questionnaire used in this study is presented as Appendix S1. Hepatitis B vaccination history was obtained through self‐report and verified against vaccination cards where available. Where vaccination cards were unavailable, vaccination status was based on participants′ self‐reported history.

### 2.6. HBV Surface Antigen (HBsAg) Testing

Following the interviews, on‐site testing was conducted using finger‐prick blood samples. The fingertip of each participant was thoroughly disinfected with 70% ethanol and allowed to dry completely before performing the finger prick using disposable sterile lancets. This allowed for collection of whole blood samples for immediate testing.

Serological testing for HBsAg was performed on‐site using the Hightop HBV One Step Rapid Test (Hightop Biotech Co. Ltd., Qingdao, China) according to the manufacturer′s instructions. However, rapid diagnostic tests (RDTs) may have lower sensitivity compared with ELISA‐based assays, potentially leading to underestimation of true prevalence. The whole blood sample obtained from finger prick was applied directly to the test device, and buffer solution was added as specified in the manufacturer′s protocol with results read within 15–20 min. All test sample results were then confirmed by a second person for positivity or negativity. Quality control measures were implemented throughout the testing procedures, including the use of positive and negative controls with each new batch of test kits to ensure accuracy and reliability of results.

The Hightop One Step Rapid Test was used for HBsAg detection because of its practicality in large field‐based studies where laboratory infrastructure is limited. HBsAg RDTs have shown high diagnostic accuracy in meta‐analysis, with pooled sensitivity around 90% and specificity around 99.5% compared with immunoassays [19]. Its ability to provide immediate results was crucial in this study, as it allowed on‐site counseling, referral of positive cases, and minimized loss to follow‐up. The Hightop One Step Rapid Test was used for HBsAg detection because of its practicality for large‐scale field‐based studies where laboratory infrastructure is limited. We could not identify reliable independent diagnostic sensitivity, specificity, or validation data specific to the Hightop One Step Rapid Test used in this study. Therefore, the available pooled evidence from a systematic review and meta‐analysis of HBsAg RDTs, which reported pooled sensitivity of approximately 90% and specificity of 99.5% compared with immunoassays, was used as supportive evidence for the diagnostic performance of rapid HBsAg testing. However, these pooled estimates do not represent independent validation of the specific Hightop assay used in this study, and the possibility of false‐negative or false‐positive results cannot be excluded.

### 2.7. Data Analysis

Data collected were entered onto Microsoft Excel sheet and exported to IBM SPSS Version 26.0 and GraphPad Prism Version 8.0 for analysis. Categorical data were presented as frequencies (proportions). The overall prevalence of HBV infection and occupation‐specific prevalence rates were calculated. Chi‐square tests were used to examine associations between independent variables and Hepatitis B infection status. Logistic regression analysis was performed to identify independent predictors of Hepatitis B infection. Crude odds ratios (cOR) and adjusted odds ratios (aOR) with 95% confidence intervals (CI) were reported. A *p* value < 0.05 was considered statistically significant. Multicollinearity was assessed, and no significant collinearity was observed (VIF < 2).

## 3. Results

### 3.1. Sociodemographic and Behavioral Factors Associated With Hepatitis B Infection

The age group with the highest frequency was ≤ 30 years (159, 32.5%). Most of the participants were female (279, 57.1%) and were married (291, 59.5%). Majority of them were Christians (334, 68.3%), had basic education (240, 49.1%), and have stayed in urban areas for greater part of their lives (358, 73.2%). Also, more than half (307, 62.8%) of them had not received any education on Hepatitis B infection. Majority of them (361, 73.4%) had never contracted STIs, and most of them (369, 75.5%) had their first sexual intercourse ≥ 18 years old. Over two‐third of them (437, 89.4%) had never received blood transfusion and did not have tattoos (451, 92.2%). Additionally, most of them did not have close person living with Hepatitis B (420, 85.9%) and had had their fingernails/toenails clipped by commercial nail clippers before (349, 71.4%), and majority of them had multiple sexual partners (351, 71.8%). Most (306, 62.6%) of them had not taken any shot of the Hepatitis B vaccine (Table 1).

**Table 1:** Sociodemographic and behavioral factors associated with Hepatitis B infection.

Variables	489 (100%), *n* (%)	Hepatitis B infection status		*p*
		Negative (453, 92.64%), *n* (%)	Positive (36, 7.36%), *n* (%)	
Age (in years)				0.068
≤ 30	159 (32.5)	151 (95.0)	8 (5.0)	
31–40	105 (21.5)	94 (89.5)	11 (10.5)	
41–50	118 (24.1)	105 (89.0)	13 (11.0)	
≥ 51	107 (21.9)	103 (86.3)	4 (3.7)	
Gender				**0.022**
Female	279 (57.1)	265 (95.0)	14 (5.0)	
Male	210 (42.9)	188 (89.5)	22 (10.5)	
Marital status				0.628
Cohabiting	55 (11.2)	49 (89.1)	6 (10.9)	
Divorced	12 (2.5)	11 (91.7)	1 (8.3)	
Married	291 (59.5)	269 (92.4)	22 (7.6)	
Single	115 (23.5)	108 (93.9)	7 (6.1)	
Widowed	16 (3.3)	16 (100.0)	0 (0.0)	
Religion				0.216
Christian	334 (68.3)	312 (93.4)	22 (6.6)	
Islamic	139 (28.4)	125 (89.9)	14 (10.1)	
Others	16 (3.3)	16 (100.0)	0 (0.0)	
Educational level				0.674
No formal education	60 (12.3)	57 (95.0)	3 (5.0)	
Basic	240 (49.1)	219 (91.3)	21 (8.8)	
Secondary	151 (30.9)	141 (93.4)	10 (6.6)	
Tertiary	38 (7.8)	36 (94.7)	2 (5.3)	
Residence				0.515
Rural	127 (26.0)	116 (91.3)	11 (8.7)	
Urban	362 (74.0)	337 (93.1)	25 (6.6)	
Work type				0.189
Butcher and meat seller	77 (15.7)	67 (87.0)	10 (13.0)	
Driver and bus conductor	30 (6.1)	27 (90.0)	3 (10.0)	
Fish monger	41 (8.8)	40 (97.6)	1 (2.4)	
Food vendor	105 (21.5)	97 (92.7)	8 (7.6)	
General goods store keeper	87 (17.8)	85 (97.7)	2 (2.3)	
Market porter (kayaye)	52 (10.6)	48 (92.3)	4 (7.7)	
Tailor and seamstress	97 (19.8)	89 (91.8)	8 (8.2)	
Have you received education on Hepatitis B before?				0.886
No	307 (62.8)	284 (92.5)	23 (7.5)	
Yes	182 (37.2)	169 (92.9)	13 (7.1)	
To the best of your knowledge, have you ever had STDs before?				0.535
No	361 (73.4)	336 (93.1)	25 (6.9)	
Yes	128 (26.2)	117 (91.4)	11 (8.6)	
At what age did you have your first sexual intercourse (in years)				0.689
< 18	113 (23.1)	106 (93.8)	7 (6.2)	
≥ 18	369 (75.5)	341 (92.4)	28 (7.6)	
Unwilling to disclose	7 (1.4)	6 (85.7)	1 (14.3)	
Have you been transfused with blood before?				0.307
No	437 (89.4)	403 (92.2)	34 (7.8)	
Yes	52 (10.6)	50 (96.2)	2 (3.8)	
Do you have a tattoo on you?				**0.007**
No	451 (92.2)	422 (93.6)	29 (6.4)	
Yes	38 (7.8)	31 (81.6)	7 (18.4)	
Have fingernails/toenails clipped by commercial nail clippers before?				0.617
No	140 (28.6)	131 (93.6)	9 (6.4)	
Yes	349 (71.4)	322 (92.3)	27 (7.7)	
Do you have a close person who has Hepatitis B infection?				0.591
No	420 (85.9)	388 (92.4)	32 (7.6)	
Yes	69 (14.1)	65 (94.2)	4 (5.8)	
Sexual partners				0.656
Multiple	351 (71.8)	324 (92.3)	27 (7.7)	
Single/none	138 (28.2)	129 (93.5)	9 (6.5)	
Hepatitis B vaccination status				**0.021**
No	306 (62.6)	227 (90.5)	29 (9.5)	
Yes	183 (37.4)	176 (96.2)	7 (3.8)	

*Note:* Data is presented as frequency (percentage). A *p* value of < 0.05 was considered statistically significant for Hepatitis B infection status. The bold values indicate *p* values which are statistically significant for Hepatitis B infection status.

There was significant association between gender (*p* = 0.0220), presence of tattoos (*p* = 0.007), Hepatitis B vaccination status (*p* = 0.021), and Hepatitis B infection. However, age (*p* = 0.068), marital status (*p* = 0.628), religion (*p* = 0.216), educational level (*p* = 0.674), residence (*p* = 0.515), work type (*p* = 0.189), awareness of Hepatitis B (*p* = 0.886), history of sexually transmitted diseases (STDs) (*p* = 0.535), age at first sexual intercourse (*p* = 0.689), history of blood transfusion (*p* = 0.307), experiences with fingernail or toenail clipping (*p* = 0.617), having close person with Hepatitis B infection (*p* = 0.591), sexual partners (*p* = 0.656), and Hepatitis B vaccination status (*p* = 0.021) were not significantly associated with Hepatitis B infection status (Table 1).

### 3.2. Prevalence of HBV Infection Among the Study Respondents, Disaggregated by Number of Vaccine Doses Received

**Figure 1:**
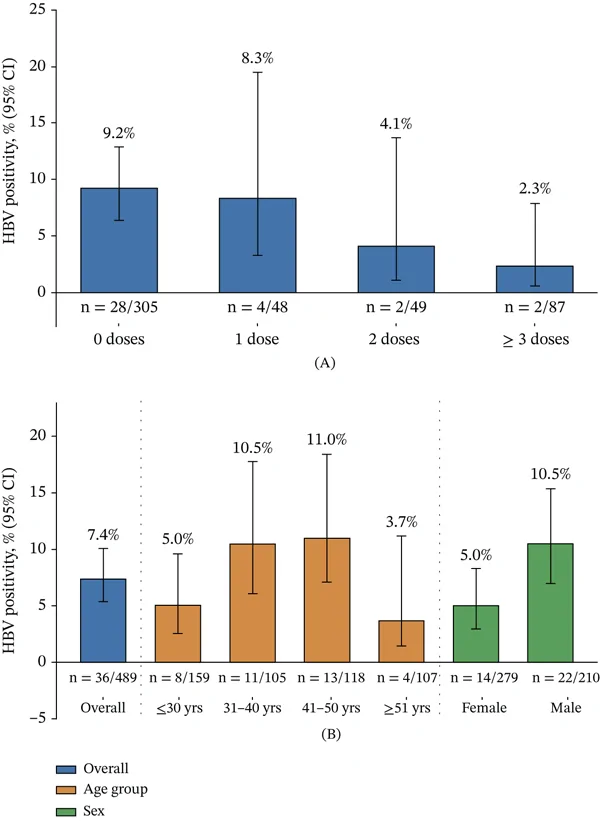
Prevalence of Hepatitis B virus (HBV) infection among the study respondents, disaggregated by number of vaccine doses received.

Figure 1 shows the prevalence of HBV infection among the study respondents, disaggregated by number of vaccine doses received. Figure 1A shows a clear inverse relationship between the number of Hepatitis B vaccine doses received and HBV positivity. Prevalence was highest among unvaccinated participants at 9.2% (95% CI: 6.4%–12.9%) and declined progressively to 8.3% (95% CI: 3.3%–19.6%) among those who had received one dose, 4.1% (95% CI: 1.1%–13.7%) among those with two doses, and 2.3% (95% CI: 0.6%–8.0%) among those who had completed three or more doses (Figure 1A). Figure 1B shows the overall prevalence of HBV infection alongside prevalence stratified by age group and sex. The overall prevalence of HBV infection among study participants was 7.4% (95% CI: 5.4%–10.0%). By age group, prevalence was lowest among those aged ≤ 30 years at 5.0% (95% CI: 2.6%–9.6%) and those aged ≥ 51 years at 3.7% (95% CI: 1.5%–9.2%), and highest among the middle‐aged groups at 10.5% (95% CI: 6.0%–17.8%) among those aged 31–40 years and 11.0% (95% CI: 6.6%–17.9%) among those aged 41–50 years, although the overlapping confidence intervals across age groups suggest this pattern should be interpreted as a trend rather than a statistically confirmed difference. By sex, HBV prevalence was more than twice as high among males, at 10.5% (95% CI: 7.0%–15.4%), compared with females, at 5.0% (95% CI: 3.0%–8.2%) (Figure 1B).

### 3.3. Univariate and Multivariate Logistic Regression Analysis for the Factors Associated With Hepatitis B Infection in Kejetia

Table 2 shows the univariate and multivariate logistic regression analysis for the factors associated with Hepatitis B infection among the study respondents. Compared with participants aged ≤ 30 years, those aged 41–50 years were 3.8 times more likely to have Hepatitis B infection (aOR = 3.799; 95% CI: 1.179–12.238; *p* = 0.025). Participants with tattoos had approximately 3.5‐fold higher odds of Hepatitis B infection than those without tattoos (aOR = 3.524; 95% CI: 1.185–10.498; *p* = 0.023). Likewise, participants who had not received the Hepatitis B vaccine were four times more likely to have Hepatitis B infection than those who had been vaccinated (aOR = 4.001; 95% CI: 1.511–10.593; *p* = 0.005) (Table 2).

**Table 2:** Univariate and multivariate logistic regression analysis for the factors associated with Hepatitis B infection in Kejetia.

Variables	cOR	aOR	*p*
Age (in years)			
≤ 30	1.00		
31–40	2.209 (0.857–5.691)	2.519 (0.879–7.222)	0.086
41–50	2.237 (0.936–5.836)	3.799 (1.179–12.238)	**0.025**
≥ 51	0.733 (0.215–2.498)	0.954 (0.239–3.811)	0.947
Sex			
Female	1.00		
Male	2.215 (1.105–4.441)	1.800 (0.506–6.404)	0.364
Educational level			
No formal education	0.947 (0.151–5.948)	0.418 (0.054–3.220)	0.402
Basic	1.726 (0.388–7.679)	0.901 (0.168–4.823)	0.903
Secondary	1.277 (0.268–6.085)	1.087 (0.204–5.790)	0.922
Tertiary	1.00		
Residence			
Rural	1.278 (0.610–2.679)	1.548 (0.641–3.738)	0.332
Urban	1.00		
Work type			
Butcher and meat seller	6.343 (1.344–29.933)	3.554 (0.551–22.912)	0.182
Driver and bus conductor	4.722 (0.749–29.759)	2.261 (0.268–19.080)	0.454
Fish monger	1.062 (0.094–12.065)	1.043 (0.080–13.686)	0.974
Food vendor	3.505 (0.724–16.920)	4.012 (0.761–21.144)	0.101
Market porter (kayaye)	3.542 (0.625–20.055)	3.378 (0.405–28.133)	0.260
Tailor and seamstress	3.820 (0.789–18.505)	2.916 (0.484–17.581)	0.243
General goods store keeper	1.00		
Have you received education on Hepatitis B before?			
No	1.00		
Yes	0.950 (0.469–1.925)	1.419 (0.607–3.313)	0.419
To the best of your knowledge, have you ever had STDs before?			
No	1.00		
Yes	1.264 (0.603–2.648)	0.846 (0.358–2.002)	0.704
At what age did you have your first sexual intercourse (in years)			
< 18	0.396 (0.042–3.716)	0.199 (0.016–2.410)	0.204
≥ 18	0.493 (0.057–4.237)	0.206 (0.019–2.274)	0.204
Unwilling to disclose	1.00		
Have you been transfused with blood before?			
No	1.00		
Yes	0.474 (0.111–2.033)	0.417 (0.085–2.037)	0.280
Do you have a tattoo on you?			
No	1.00		
Yes	3.286 (1.333–8.101)	3.524 (1.185–10.498)	**0.023**
Have fingernails/toenails clipped your nails before?			
No	1.00		
Yes	1.220 (0.559–2.666)	1.180 (0.484–2.875)	0.716
Do you have a close person who has Hepatitis B infection?			
No	1.00		
Yes	0.746 (0.255–2.180)	1.653 (0.516–5.291)	0.397
How many different sexual partners have you had since childhood/lifetime?			
Multiple	1.194 (0.547–2.610)	1.195 (0.434–3.285)	0.731
Single/none	1.00		
Vaccination status			
No	2.632 (1.129–6.138)	4.001 (1.511–10.593)	**0.005**
Yes	1.00		

*Note:* 1.00, reference. Binary logistic regression analysis was performed to obtain odds ratios. A *p* value of < 0.05 was considered statistically significant for Hepatitis B infection status. The bold values indicate *p* values which are statistically significant for Hepatitis B infection status.

Abbreviations: aOR, adjusted odds ratio; CI, confidence interval; cOR, crude odds ratio.

## 4. Discussion

This study examined the prevalence and predictors of HBV infection among sellers and workers at the Kejetia Market, Ghana′s largest commercial hub. The overall prevalence of HBV infection in the study population was 7.36% (Figure 1A). This prevalence falls within the intermediate‐to‐high endemic range as defined by WHO [20]. This highlights the fact that HBV infection is still a major public health concern in Ghana. The finding is consistent with 7.5% HBV prevalence reported among blood donors in Volta Region of Ghana [21], 7.2% prevalence among adults in Northern Ghana [22], and 7.88% in Southern Ethiopia [23]. The current finding is slightly lower than the pooled HBV prevalence of 8.36% among adults in Ghana by Abesig et al. [4] and crude prevalence of 8.48% by Nartey et al. [8], suggesting that the Kejetia Market population mirrors the broader regional epidemiological pattern. In contrast, the current prevalence is significantly lower than the prevalence of 15.6% by Helebge et al. [24] among market women in the northern part of the country. It is important to note that this study was done about 5 years prior to the current study. The observed difference in prevalence may reflect variations in study population characteristics, geographical setting, study period, vaccination coverage, and other unmeasured factors. However, these explanations cannot be established from the present cross‐sectional study and should therefore be interpreted cautiously. Nonetheless, this sharp contrast in the prevalence rate could be due to differences in geographical locations and lifestyles as well. It is recommended for further studies to explore this occurrence. Still, the finding is higher than prevalence studies reported in other parts of the country, such as 4.4% among pregnant women in Eastern Ghana [25], 5.9% among healthcare workers in Southern Ghana [7], and 2.4% among pregnant women in Volta Region of Ghana [26]. The HBV prevalence in the current study is comparatively higher than that of the 6.9% for blood donors and 5.9% for pregnant women reported for Africa in current systematic review and meta‐analysis [27, 28]. Again, the current prevalence is more than double the global HBV prevalence (3.2%) recently reported [29], indicating a high community burden in Kejetia adult population. This emphasizes that more efforts are required in Ghana to achieve the WHO′s goal to eliminate HBV infection by 2030 [30].

Being female appeared to be protective against HBV infection, as females were almost 0.5 times less likely to be infected with Hepatitis B compared with males (Table 2). This is consistent with prior reports from other studies, where men often exhibit higher HBsAg prevalence compared with women [31–33, 32]. Possible explanations include gender differences in health‐seeking behavior, occupational exposure, and engagement in higher‐risk practices such as unsafe sexual activity or alcohol use, which may increase susceptibility to HBV infection. Additionally, men are less likely to participate in preventive health programs, including vaccination campaigns, potentially contributing to this disparity [34].

Not having a tattoo was associated with a lower risk of HBV infection. Individuals without tattoos were almost 0.3 times as likely to contract Hepatitis B compared with those with tattoos (Table 2). Tattooing, especially when performed under unregulated or unsafe conditions, can facilitate transmission through contaminated needles and equipment due to poor sterilization. Other studies have similarly reported associations between tattooing, body scarification, and HBV seropositivity [35–38]. Given the cultural and social significance of tattoos and related practices in many African communities, public health strategies should emphasize the importance of safe procedures and infection prevention practices in both formal and informal tattoo settings.

Finally, vaccination uptake was inversely associated with HBV infection, underscoring the protective role of full adherence to the Hepatitis B vaccination schedule. When compared with those who have been vaccinated, those who have not been vaccinated against Hepatitis B had 3.4‐fold risk of getting infected with HBV. This finding supports the protective effect of the vaccine which has also been shown in Figure 1B, where there was a noticeable decreasing prevalence of HBV positivity with increasing number of vaccine shots [39]. Participants who did not complete the recommended three‐dose series were more likely to be HBsAg‐positive, in line with previous studies demonstrating that incomplete vaccination compromises long‐term immunity [40, 41]. In Ghana, despite the introduction of the HBV vaccine into the Expanded Programme on Immunization (EPI) in 2002, adult coverage remains low, and poor compliance with vaccination schedules has been reported among healthcare workers and the general population [13, 42]. Our findings highlight the urgent need to strengthen vaccination programs and ensure the availability and affordability of vaccines. In Ghana, strengthening adult Hepatitis B vaccination programs and ensuring completion of the recommended vaccination schedule should remain the priority. Although postvaccination serological testing may provide useful information on immune response, routine testing may not be feasible or cost‐effective in low‐resource informal‐sector settings and could therefore be considered selectively where clinically or programmatically indicated.

Although occupational category was not found to be a statistically significant predictor of HBV infection in this study, variations in prevalence were observed across different work groups. The highest prevalence was recorded among butchers and meat sellers (13.0%) and drivers/bus conductors (10.0%) [4], whereas relatively lower rates were seen among fishmongers (2.4%) and general goods storekeepers (2.3%). Tailors/seamstresses, market porters, and food vendors exhibited intermediate prevalence levels of 8.2%, 7.7%, and 7.6%, respectively. These findings, though not statistically significant, are noteworthy as they may reflect differences in occupational exposures and workplace practices. For instance, butchers and drivers may be at increased risk of accidental cuts, exposure to blood, or unregulated sharp object use, which are recognized routes of HBV transmission. By contrast, fishmongers and storekeepers may face fewer such exposures in the course of their work. The absence of statistical significance could be due to overlapping behavioral risk factors across occupations, relatively small subgroup sample sizes that limited statistical power, or the influence of protective factors such as vaccination in some occupational groups. Nonetheless, these occupational prevalence patterns provide useful insights into possible priority groups for HBV awareness, screening, and vaccination interventions within informal work settings.

Together, these findings have important public health implications. The persistence of intermediate‐to‐high HBV prevalence among market workers, a group that reflects the general adult population, suggests that Ghana remains far from achieving the WHO′s target of reducing Hepatitis B incidence by 90% and mortality by 65% by 2030 [30]. Targeted interventions such as increasing awareness of safe tattooing practices, promoting male‐focused screening and vaccination campaigns, and improving compliance with vaccination schedules are essential steps toward reducing HBV burden in Ghana.

In contrast to findings from previous studies that have consistently identified blood transfusion, early sexual debut, multiple sexual partners, and household exposure to HBV‐positive individuals as significant risk factors for HBV infection [23, 32, 43, 44], our study did not demonstrate statistically significant associations for these variables. Several factors may explain these observations. First, the risk of transfusion‐associated HBV transmission has declined substantially in Ghana following the introduction of routine HBV screening of donated blood, thereby reducing its contribution as a transmission route in contemporary settings [45, 46]. Second, although sexual transmission remains an established pathway, our study population was largely older adults, many of whom may already have acquired immunity through prior vaccination, reducing the relative impact of sexual behaviors on current infection status. Additionally, the reliance on self‐reported data for sexual history and family infection status may have introduced recall or social desirability bias, leading to underreporting and consequent dilution of associations. Finally, the occupational nature of the study population suggests that practices such as tattooing may play a more dominant role than traditional household or sexual risk factors, which could explain why these factors lost significance in multivariable models.

### 4.1. Policy and Public Health Implications

The findings of this study have important implications for Hepatitis B control strategies in Ghana. First, the high prevalence among informal workers highlights the need for targeted adult vaccination campaigns, particularly outside formal healthcare settings. Second, occupational health education and safety training should be integrated into market environments to reduce exposure to blood and sharps. Third, regulation and monitoring of informal tattooing practices are essential to minimize transmission risks. Finally, male‐focused screening and vaccination interventions should be prioritized, given their higher observed risk.

### 4.2. Strengths and Limitations

This study has some limitations. Being cross‐sectional in nature, it cannot establish causality between the predictors and HBV infection. Additionally, recall bias and social desirability bias could affect the accuracy of self‐reported data. Also, the use of RDTs, although practical in field settings, may have lower sensitivity and specificity compared with ELISA or molecular assays. Again, the study was conducted at a single site (Kejetia Market), which may limit generalizability to other populations. Again, vaccination status and behavioural risk factors were self‐reported and may therefore be subject to recall and social desirability bias. Additionally, only HBsAg was assessed; anti‐HBs and anti‐HBc were not measured, limiting our ability to determine previous HBV exposure and distinguish naturally acquired from vaccine‐induced immunity. Future studies should consider incorporating a comprehensive HBV serological panel alongside documented vaccination history, where feasible. Nonetheless, the findings provide valuable insight into HBV epidemiology in an important urban working population and contribute to the evidence base guiding HBV prevention and control strategies in Ghana. The findings provide evidence to guide targeted interventions in informal sector populations, which are often underrepresented in surveillance data. Also, the use of stratified random sampling enhanced the representativeness of the study population and minimized selection bias, thereby improving the generalizability of the findings.

## 5. Conclusion

HBV infection remains a significant public health challenge among Kejetia Market workers, with a prevalence of 7.36%, reflecting intermediate‐to‐high endemicity. Female gender, absence of tattooing, and Hepatitis B vaccination were associated with lower odds of HBV infection. Targeted adult vaccination, promotion of safe tattooing practices, and focused HBV prevention interventions may help reduce transmission among informal‐sector workers. Further studies in other settings are needed to determine the generalizability of these findings to the wider Ghanaian population.

**Nomenclature**
aOR: adjusted odds ratio
CHRPE: Committee on Human Research, Publication and Ethics
CI: confidence interval
cOR: crude odds ratio
EPI: Expanded Programme on Immunization
HBsAg: Hepatitis B surface antigen
HBV: Hepatitis B virus
HCV: Hepatitis C virus
HIV: human immunodeficiency virus
NVHCP: National Viral Hepatitis Control Programme
RDT: rapid diagnostic test
SDG: Sustainable Development Goal
SPSS: Statistical Package for the Social Sciences
SSA: sub‐Saharan Africa
STI: sexually transmitted infection
WHO: World Health Organization

## Author Contributions

Investigation/data collection: De‐Graft Kwaku Ofosu Boateng, Michael Agyemang Obeng, Joshua Kofi Attah, Abdul Latif Koney Shardow, Clinton Owusu Boateng, Daniel Kobina Okwan, Senam Yawa Nunamey Ahadzie, Pius Takyi, Godfred Yawson Scott, Akwasi Amponsah Abrampah, Felix Kusi Boakye, Patrick Oduro, Daniel Dzemedo Nouwati, Kofi Bonsu Amankwah, Reem Mahmoud Hassanin, Mohammed Mahi Adams, and Abu Abudu Rahamani. Data curation: De‐Graft Kwaku Ofosu Boateng, Michael Agyemang Obeng, Joshua Kofi Attah, Abdul Latif Koney Shardow, Clinton Owusu Boateng, Daniel Kobina Okwan, Senam Yawa Nunamey Ahadzie, Pius Takyi, Godfred Yawson Scott, Akwasi Amponsah Abrampah, Felix Kusi Boakye, Patrick Oduro, Daniel Dzemedo Nouwati, Kofi Bonsu Amankwah, Reem Mahmoud Hassanin, Mohammed Mahi Adams, and Abu Abudu Rahamani. Formal analysis: Godfred Yawson Scott, De‐Graft Kwaku Ofosu Boateng, Michael Agyemang Obeng, Clinton Owusu Boateng, and Daniel Kobina Okwan. Resources/materials: De‐Graft Kwaku Ofosu Boateng, Michael Agyemang Obeng, Joshua Kofi Attah, Abdul Latif Koney Shardow, Clinton Owusu Boateng, Daniel Kobina Okwan, Senam Yawa Nunamey Ahadzie, and Pius Takyi. Supervision: De‐Graft Kwaku Ofosu Boateng, Michael Agyemang Obeng, Daniel Kobina Okwan, and Abu Abudu Rahamani. Project administration: De‐Graft Kwaku Ofosu Boateng, Michael Agyemang Obeng, Joshua Kofi Attah, Clinton Owusu Boateng, Daniel Kobina Okwan, and Abu Abudu Rahamani. Writing—original draft: De‐Graft Kwaku Ofosu Boateng, Michael Agyemang Obeng, Joshua Kofi Attah, Abdul Latif Koney Shardow, Clinton Owusu Boateng, and Daniel Kobina Okwan. Writing—review and editing: De‐Graft Kwaku Ofosu Boateng, Michael Agyemang Obeng, Joshua Kofi Attah, Abdul Latif Koney Shardow, Clinton Owusu Boateng, Daniel Kobina Okwan, Senam Yawa Nunamey Ahadzie, Pius Takyi, Godfred Yawson Scott, Akwasi Amponsah Abrampah, Felix Kusi Boakye, Patrick Oduro, Daniel Dzemedo Nouwati, Kofi Bonsu Amankwah, Reem Mahmoud Hassanin, Mohammed Mahi Adams, and Abu Abudu Rahamani. Visualization: De‐Graft Kwaku Ofosu Boateng, Michael Agyemang Obeng, Joshua Kofi Attah, Abdul Latif Koney Shardow, Clinton Owusu Boateng, Daniel Kobina Okwan, Senam Yawa Nunamey Ahadzie, Pius Takyi, Godfred Yawson Scott, and Abu Abudu Rahamani. Validation: De‐Graft Kwaku Ofosu Boateng, Michael Agyemang Obeng, Joshua Kofi Attah, Clinton Owusu Boateng, Daniel Kobina Okwan, Pius Takyi, Godfred Yawson Scott, and Abu Abudu Rahamani.

## Funding

No funding was received for this manuscript.

## Ethics Statement

Ethical approval was obtained from the Committee on Human Research, Publication and Ethics (CHRPE) of the School of Medical Sciences, KNUST with Approval Number CHRPE/AP 180/24. Kejetia management gave site approval to conduct the study at the market. Written informed consent was obtained from all participants after explaining the purpose, procedures, benefits, and potential risks of the study in their preferred language.

Confidentiality was maintained by using unique identification codes in place of personal identifiers on all study documents. Participants found to be HBsAg‐positive were counseled and referred to appropriate healthcare facilities for further evaluation and management. The ethical compliance was done in accordance with the Declaration of Helsinki.

## Consent

The authors have nothing to report.

## Conflicts of Interest

The authors declare no conflicts of interest.

## Supporting information


**Supporting Information 1** Additional supporting information can be found online in the Supporting Information section. Appendix S1: Kejetia viral Hepatitis B study questionnaire_KJM1.

## Data Availability

The data that support the findings of this study are openly available in Dryad at 10.5061/dryad.cjsxksnkd.
