# Supplementary material for: Prevalence and Predictors of Hepatitis B Virus Infection Among Sellers and Workers at West Africa′s Largest Market, Kejetia, Ghana: A Cross‐Sectional Study
Source: Biomed Res Int. 2026 Sep 23;2026:1412674. doi: 10.1155/bmri/1412674 (PMC13598932; doi:10.1155/bmri/1412674)
Supplement: Supplementary file 1 [file BMRI-2026-1412674-s001.docx]

KEJETIA VIRAL HEPATITIS B STUDY QUESTIONNAIRE_KJM1

You have been invited in a research study that seeks to assess prevalence and predictors of hepatitis B viral infection among sellers and workers at Kejetia. Please note that by completing this questionnaire you are voluntarily agreeing to participate in this research study. You will remain anonymous and your data will be treated with great confidentiality at all times.

The researchers will be glad if you could complete the questionnaire in full. Please tick the appropriate box (es) and/or fill the blank spaces provided.

**1. Have you ever heard of hepatitis B infection?**

Yes

No

**2. If yes, what is your source of information on hepatitis B?**

Media

School

Church

Friends

Hospital

Other:

**3. What is the main target organ for hepatitis B?**

Leg

Kidney

Liver

Intestines

Other:

**4. Which of the following are transmission routes of hepatitis B?**

contaminated food and water

mother-to-child

shaking hands with infected persons

hugging

through sex

Other:

**5. Which group of people are encouraged to take the hepatitis B vaccine?**

Those who are infected with hepatitis B

Those who do not yet have the hepatitis B infection

Other:

**6. Do you know if there is vaccine available for hepatitis B?**

Yes

No

Other:

**7. Have you taken Hepatitis B vaccine before?**

Yes

No

Other:

**8. If no, (not vaccinated), will you take the hepatitis B vaccine if it were given free of charge?**

Yes

No

Other:

**8. If yes, how many doses of the hepatitis B vaccine have you taken?**

0

1

2

3

≥4

**10. If vaccinated, when was the last time you took Hepatitis B vaccine?**

18. If vaccinated, do you have the document or yellow card showing proof of vaccination?

Yes

No

Other:

**11. Do you have a close person who has HBV infection?**

Yes

No

Other:

**12. Where you have stayed for greater part of your life, will you consider that place to be?**

Rural

Urban

Other:

**13. How many different sexual partners have you had since childhood / lifetime?**

…………………………………………..

**14. To the best of your knowledge, have you ever had STDs before, for example HIV, Hepatitis, Syphilis, Gonorrhoea or Chlamydia?**

Yes

No

Other:

**15. At which age did you have your first sexual intercourse?**

----------------------------------

**16. Have you been given blood transfusion before?**

Yes

No

Other:

**17. If yes, how many times (number of bags or pints)?**

………………………………………

**18. Do you have tattoo on you?**

Yes

No

Other:

**19. The hepatitis B testing and vaccination at the moment cost 60.00 for first shot and 30.00 for 2nd and third each; How expensive do you think is the testing and vaccination of Hepatitis B?**

Free

Reasonable

Somewhat expensive

Expensive

Don't know

Other:

**20. Have commercial fingernails / toenails clippers clipped your nails for you before?**

Yes

No

**21. If yes, how many times?**

………………………………..

**22. Have you ever had a cut from the cutting of nails by fingernails / toenails clippers before?**

Yes

No

Other:

**23. Have you had any extra piercings aside the one you had as a child?**

Yes

No

**24. Have you injected intravenous drug before?**

Yes

No

**25. Have you received education or training on Hepatitis B before?**

Yes

No

Other:

**26. If you are a worker (or seller) at Kejetia, does your work here sometimes result in a cut or injury to you?**

Yes

No

**27. If yes, how many times have you got a cut or injury from your work here (estimate)?**

**……………………………………………………..**

**28. For both sellers (Kejetia workers) and buyers, have you gotten a cut or injury from Kejetia here before (cut from any activity at all, once it is at Kejetia here)?**

Yes

No

**29. If yes, how many times (estimate)?**

**…………………………………………………………**

**30. If yes, what led to your cut or injury at Kejetia here?**

…………………………………………………………..

**31. If seller or worker at Kejetia, how many times do you wash your hands within a day at work? (eg. 5 times a day)**

………………………………………………………………..

**32. Do you use soap when washing hands or only water?**

………………………………………………………………..

Only water mostly

Soap with water mostly

Other:

**33. If seller or worker at Kejetia, have you attended education program on hepatitis B at your workplace before?**

Yes

No

Other:

**34. As an individual, will you attend it if education program on Hepatitis B is organised for people at your workplace?**

Yes

No

Other:

**DEMOGRAPHICS**

**1. Sex**

Male

Female

**2. Age**

**3. Marital status**

Divorced

Single

Married

Widowed

Cohabiting

**4. Religion**

Islamic

Christian

Traditional

Other:

**5. Educational level**

None

Basic

Secondary

Certificate

Diploma

HND

1st Degree

Master's degree

PhD

**6. Participant ID**

………………………….

**14. Where do you stay (ie where you commute daily to Kejetia for work)?**

……………………………………………………..

**15. Which region is Q14 found?**

………………………………………………………..

**17. Profession categorization for participants**

Tailors & seamstress

Butchers & Meat Sellers

Barbers and Hairdressers

Waste collectors and cleaners

Fish mongers

Market porters (Kayayei)

Drivers & Mates

Food Vendors

Mobile workers (Hawkers)

Buyers

Cloth and Textile Sellers

Other:

**16. Initials of investigator in the order of first, middle and surname (eg. MAO for Michael Agyemang Obeng)**

………………………………………………………..
